# Supplementary material for: Micronutrients Affect Expression of Induced Resistance Genes in Hydroponically Grown Watermelon against Fusarium oxysporum f. sp. niveum and Meloidogyne incognita
Source: Pathogens. 2022 Sep 30;11(10):1136. doi: 10.3390/pathogens11101136 (PMC9608861; doi:10.3390/pathogens11101136)
Supplement: Supplementary file 1 [file pathogens-11-01136-s001.zip › pathogens-1891427-supplementary.pdf]

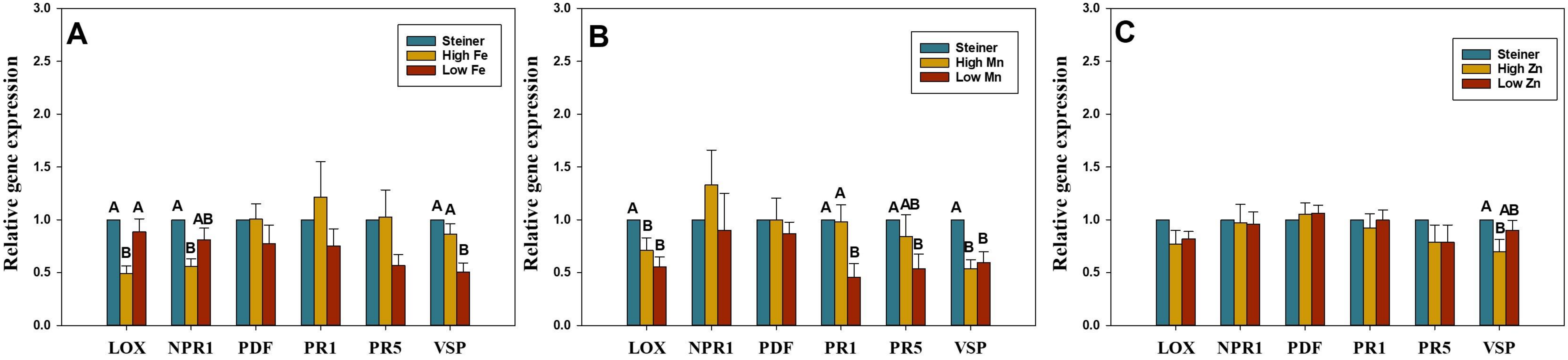

Figure S1. Relative expression of *NPR1*, *PR1*, *PR5*, *LOX*, *PDF* and *VSP* genes by qRT-PCR in watermelon leaves at 11 day-post-treatment with micronutrients A) Fe, B) Mn, and C) Zn via hydroponics system. Watermelon seedlings (cv. Sugar Baby; 3 weeks old) were either treated with Fe or Mn or Zn at high (3X), low (0.5X) and standard concentration (X, Steiner) for 11-days. Data are the mean fold changes ± SE in gene transcript levels in tissues from micronutrient treated plants relative to tissues from non-treated control plants in Steiner. Letters indicate a significant difference between treatments with the Tukey-Kramer test ($p<0.05$).
